# Effects of Solder Temperature on Pin Through-Hole during Wave Soldering: Thermal-Fluid Structure Interaction Analysis

**DOI:** 10.1155/2014/482363

**Published:** 2014-08-13

**Authors:** M. S. Abdul Aziz, M. Z. Abdullah, C. Y. Khor

**Affiliations:** School of Mechanical Engineering, Universiti Sains Malaysia, 14300 Nibong Tebal, Penang, Malaysia

## Abstract

An efficient simulation technique was proposed to examine the thermal-fluid structure interaction in the effects of solder temperature on pin through-hole during wave soldering. This study investigated the capillary flow behavior as well as the displacement, temperature distribution, and von Mises stress of a pin passed through a solder material. A single pin through-hole connector mounted on a printed circuit board (PCB) was simulated using a 3D model solved by FLUENT. The ABAQUS solver was employed to analyze the pin structure at solder temperatures of 456.15 K (183^°^C) < *T* < 643.15 K (370^°^C). Both solvers were coupled by the real time coupling software and mesh-based parallel code coupling interface during analysis. In addition, an experiment was conducted to measure the temperature difference (Δ*T*) between the top and the bottom of the pin. Analysis results showed that an increase in temperature increased the structural displacement and the von Mises stress. Filling time exhibited a quadratic relationship to the increment of temperature. The deformation of pin showed a linear correlation to the temperature. The Δ*T* obtained from the simulation and the experimental method were validated. This study elucidates and clearly illustrates wave soldering for engineers in the PCB assembly industry.

## 1. Introduction

The rapid development in microelectronic technologies presents additional challenges to ensure the reliability and quality of electronic assemblies. High demand in miniature, lightweight, and high-performance electronic products directs the focus toward the reliability of interconnectors between electronic components and printed circuit boards (PCBs). High solder joint reliability provides superior mechanical bonding and functionality of the component. Definitive design efforts and a proper process control of parameters such as soldering temperature are required to attain this goal. In the PCB assembly industry, wave soldering is an established metallurgical joining method using a molten solder to attach pin through-hole (PTH) components (e.g., resistor, transistor, and capacitor) to the PCB. This process enables the molten solder to fill the gap between the PTH and the board to create a mechanical joint (solder joint). The thermal and metallurgical reliability of this solder joint is one of the major issues that hinder the development of small, high-density interconnections [1]. In addition, the stresses and the displacement caused by the PTH on solder joints may result in solder joint defects. Improper control of molten solder temperature leads to solder joint fractures when mounting onto the PCB. This situation subjects the package to thermomechanical stress that exceeds the fracture strength of the solder joint. The density and the viscosity of the molten solder also significantly influence the reliability of the wave soldering [2].

The liquidus or melting temperature is identified as the most important factor in wave soldering. The eutectic temperature of Sn-Pb is 183°C, which is often regarded as the reference temperature. Efficient heat transfer ensures that the solder material melts, forms a joint, and solidifies [2]. In addition, the wetting and the flowability of the molten solder are determined by the metallurgical bonding between the PTH and the PCB. The temperature of the molten solder affects the surface tension, viscosity, and wetting behavior of the capillary flow during wave soldering. Generating wave solders at varying temperatures may influence the wetting of the PTH within the PCB, which may result in a poor solder joint. Studies on the wetting behavior and the microstructural characteristics of solder alloys were conducted by Fima et al. [3], Dariavach et al. [4], Lopez et al. [5], and Lu et al. [6]. The mechanical properties of solder alloys were also investigated [7–9]. The effects of thermal and solder joints were examined by Pang et al. [10, 11] and Michealidas and Sitaraman [12] in flip chip assemblies. Studies based on experimentation are widely conducted; however, investigations based on numerical simulations are rarely reported. Therefore, there is a wide research gap of wave soldering studies using numerical simulation technique.

In recent years, the application of virtual modeling tools has facilitated engineering and research in electronic packaging. The real time coupling method using the fluid-structure interaction (FSI) can be employed to solve reflow soldering [13], molded underfill [14], flexible PCB [15], and wire sweep [16]. In the wave soldering process, many factors such as physical design of PCB, process control (i.e., solder temperature and conveyor speed), and the material used could influence the process performance and flowability of molten solder in the PCB hole. The majority of wave soldering studies are based on experimentation, with focus on the solder joint and the materials used. Studies on the effects of temperature on capillary flow in wave soldering by using a thermal-FSI approach are still not reported in the literature. Therefore, the present study uses the thermocoupling method to examine the effects of temperature in wave soldering by considering the low (at solder melting point 456.15 K (183°C)) and extremely high process temperatures (643.15 K (370°C)). The influences of process temperature on the capillary flow, temperature distribution, deformation, and stress were investigated. Finite volume-based software (FLUENT) and a finite element solver (ABAQUS) connected by MpCCI were employed to exchange the temperature simultaneously. The predicted result was validated by the experimental result, thereby confirming the efficiency of the proposed method in solving thermal-FSI problems during wave soldering. Thus, the current modeling approach can elucidate the wave soldering process.

## 2. Governing Equations

In the wave soldering process, the molten solder fills the tiny PCB hole (consists of PTH) due to the capillary action. The phenomenon of the molten solder filling process was focused on. A single PCB hole with a PTH was considered in the simulation. Molten solder flow was analyzed by using a finite-volume-based simulation, FLUENT solver. The behavior and flow motion of molten solder were described by governing equations in FLUENT analysis. The flow of molten solder fluid was described by the conservation equation of mass, momentum, and energy, which was valid for incompressible flow as indicated below.

(1) Continuity equation:

(1) $$\begin{array}{c} \frac{\partial u}{\partial x}+\frac{\partial v}{\partial y}+\frac{\partial w}{\partial z}=0, \end{array}$$

where *u*, *v*, and *w* are the velocity in the *x*-, *y*-, and *z*-axis.

(2) Momentum equation:

 (*x*-direction)
  (2) ρ(∂u∂t+u∂u∂x+v∂u∂y+w∂u∂z) =−∂P∂x+η(∂2u∂x2+∂2u∂y2+∂2u∂z2)+ρgx,
 (*y*-direction)
  (3) ρ(∂v∂t+u∂v∂x+v∂v∂y+w∂v∂z) =−∂P∂y+η(∂2v∂x2+∂2v∂y2+∂2v∂z2)+ρgy,
 (*z*-direction)
  (4) ρ(∂w∂t+u∂w∂x+v∂w∂y+w∂w∂z) =−∂P∂z+η(∂2w∂x2+∂2w∂y2+∂2w∂z2)+ρgz,

where *ρ* is density, *t* is time, *P* is pressure, *η* is viscosity, and *g*_*x*_, *g*_*y*_, and *g*_*z*_ are gravity in the *x*-, *y*-, and *z*-axis.

(3) Energy equation:

(5) $$\begin{array}{c} \rho c_{p}\left(u\frac{\partial T}{\partial x}+v\frac{\partial T}{\partial y}+w\frac{\partial T}{\partial z}\right)=k\left(\frac{\partial^{2}T}{\partial x^{2}}+\frac{\partial^{2}T}{\partial y^{2}}+\frac{\partial^{2}T}{\partial z^{2}}\right). \end{array}$$

Molten solder at high temperature has a nearly constant viscosity. Thus, it can be described by the following.

(4) Newtonian fluid equation:

(6) $$\begin{array}{c} \eta =\frac{\tau }{\dot{\gamma }}, \end{array}$$

where *τ* is the shear stress and $\dot{\gamma }$ is the strain rate.

The basic idea of the volume of fluid (VOF) scheme is to locate and elucidate the distribution of the liquid phase by attaching each cell in the computational grid. *F* is the cell's volume fraction occupied by the solder material. Thus, the value of *F* = 1 in a cell contains only molten solder (63Sn37Pb), the value of *F* = 0 is in a grid void of molten solder, and the value 0 < *F* < 1 in “interface” cells is referred to as the melt front of molten solder material.

The governing equation of the melt front over time is expressed as

(7) $$\begin{array}{c} \frac{\partial F}{\partial t}=\frac{\partial F}{\partial t}+\nabla \cdot \left(uF\right)=0. \end{array}$$

**Figure 1:**
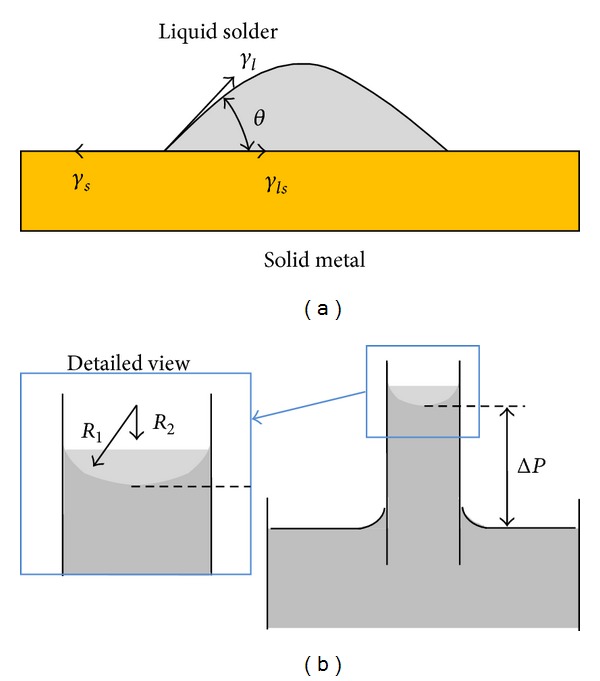
(a) Surface tension between solid surface and liquid solder and (b) capillary action of wave soldering.

To form a solder joint (Figure 1(a)), all parts must be solderable. The metal surfaces must be wetted by soldering. The wetting grade can be measured by the wetting angle according to Young's equation:

(8) $$\begin{array}{c} \gamma_{s}=\gamma_{l}\cos\theta +\gamma_{ls}, \end{array}$$

where *γ*_*s*_ is the surface tension of the solid surface, *γ*_*l*_ is the surface tension of the liquid solder, *γ*_*ls*_ is the surface tension between the solder and the solid surface, and *θ* is the boundary angle between the solder and the solid surface.

Capillary behavior is a result of the pressure differences between two sides of a curved liquid surface.  Figure 1(b) shows the capillary action during wave soldering. Pressure difference can be expressed by the law of Young and Laplace as follows:

(9) $$\begin{array}{c} \Delta P=\gamma \left(\frac{1}{R_{1}}+\frac{1}{R_{2}}\right), \end{array}$$

where Δ*P* is the pressure difference between the inside and outside of the curved liquid surface, *γ* is the liquid surface tension, and *R*_1_ and *R*_2_ are the radii of the curvature on liquid surface. Note that *R*_1_ and *R*_2_ are negative because they are outside the liquid.

## 3. Simulation Modeling

**Figure 2:**
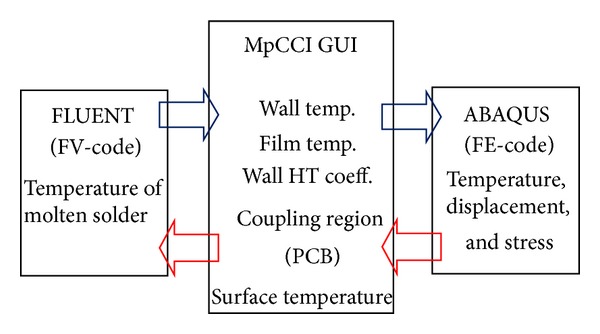
Basic concept of thermal-FSI coupling method.

**Figure 3:**
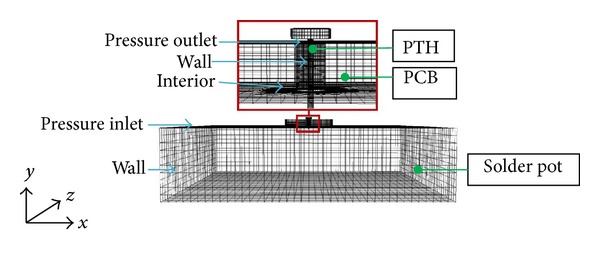
FLUENT meshed model and boundary conditions.

**Figure 4:**
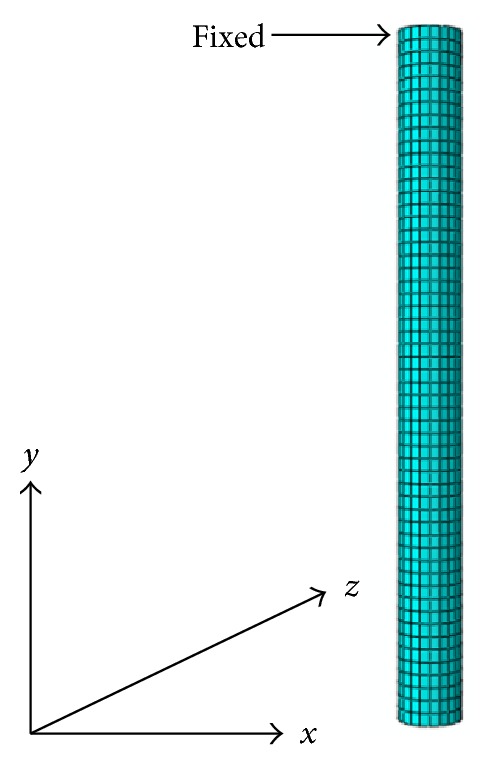
ABAQUS meshed model of PTH.

The preprocessing computational fluid dynamics software was used to construct, mesh, and set the boundary conditions of the single PTH 3D model. This model was then exported to FLUENT for fluid analysis. Structural analysis was performed using the ABAQUS structure solver. The basic concept of the thermocoupling method is shown in Figure 2. Both solvers were coupled using MpCCI, which exchanged the temperature-displacement and mesh data between FLUENT and ABAQUS [17] for thermal-FSI analysis. During the wave soldering, the PTH endures molten solder temperature. Thus, the surface of PTH was defined as a coupling region of both solvers. The optimum mesh size was obtained to minimize computational error in the grid dependency test for the fluid domain (FLUENT solver). The model with 57360 hexahedral nodes exhibited the best mesh size for fluid analysis. However, 2912 hexahedral elements were constructed in the PTH model for the structural analysis using ABAQUS software. The PCB surface, soldering pot, and PTH component were set as wall boundaries. The connection between the PCB and the soldering pot was defined as the interior; the inlet and the outlet of the model were defined as pressure boundary conditions (Figure 3). The pinhead connector was set as a fixed boundary condition and a deformable structure [18].  Figure 4 shows the meshed model and the fixed boundary condition in ABAQUS.

**Figure 5:**
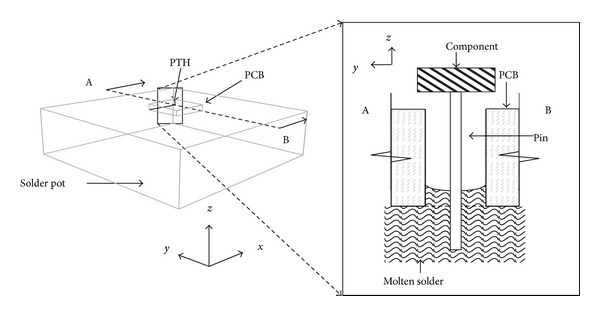
Cross-sectional view of computational domain at A-B.

The volume of fluid (VOF) model was used to track the capillary flow profile during wave soldering in the FLUENT analysis. In the VOF technique, a function of *F* (fraction) was allocated to define the fluid state. *F* = 1 when a cell is fully filled with the molten solder and *F* = 0 if a cell only contains air. A value in the range of 0 < *F* < 1 indicates that the cell is partially filled with the molten solder or the interface cell [19]. The properties of the solder material were in the range of 456.15 K < *T* < 643.15 K. Air and solder material were defined as two different phases with implicit formulations [20]. The laminar viscous model was used to describe the capillary flow during unsteady analysis. The mechanical properties of PTH were as follows: Young's modulus (*E*) = 129930 MPa, Poisson's ratio (*υ*) = 0.34, and expansion coefficient alpha (CTE) = 16.8 ppm/K [21]. The thermal properties were determined as follows: thermal conductivity = 398 W/mK, specific heat capacitance = 383 J/kg*·*K, and density = 8930 kg/m^3^ [22, 23]. The time step size was examined, with 0.0001 s [24] as the optimum for the current simulation. The current simulation focuses on the filling stage of molten solder in the PCB hole before the molten solder is solidified [25, 26]. The simulation domain was cross-sectioned by A-B (Figure 5) to observe the molten solder profile at varying solder temperatures. The simulation results were compared with the experimental results to determine the temperature difference (Δ*T*) between the top and the bottom of the PTH.

## 4. Experimental Procedure

**Figure 6:**
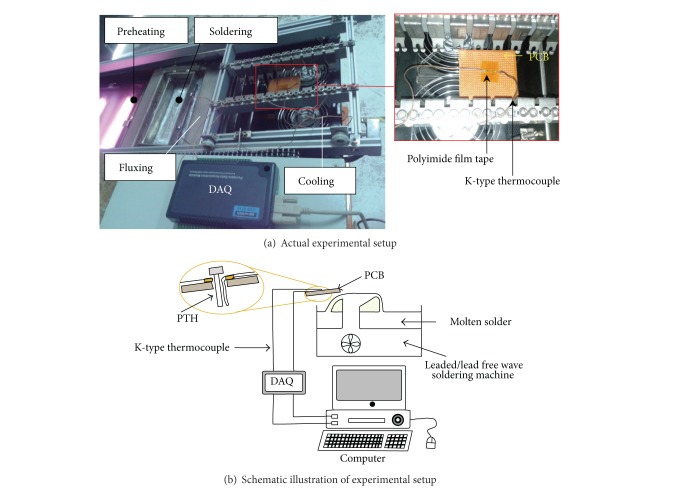
Experimental setup of wave soldering process.

An experiment was conducted to substantiate the simulation results by using two-way lead-free/leaded wave soldering machines. In the first direction, the conveyor carrying the PCB passes through the fluxing zone and stops at the preheating zone. After preheating, the conveyor moves in reverse direction; in the meantime the pump is activated and a solder fountain is generated. The PCB passes through the soldering zone and then the conveyor stops at the cooling zone. The PC-based data acquisition (DAQ) device with a high-performance monitoring and control system was used to record the temperature of the molten solder during wave soldering. The K-type thermocouples were attached to the top and the bottom of a single circular pin connector to monitor the temperature profiles during soldering. The wave soldering machine consisted of fluxing, preheating, soldering, and cooling zones (Figure 6). The soldering pot was inserted with 55 kg of Sn63Pb37 and then set to 523.15 K (250°C). The speed of the solder pump was adjusted to ensure a particularly smooth and stable solder prior to soldering. The PCB attached with thermocouples was subsequently passed through the molten solder. The temperature to which the pin was subjected during wave soldering was recorded using the DAQ. The measured experimental temperature was compared with the simulation results.

## 5. Results and Discussion

### 5.1. Experimental Validation (Δ*T* at 100% Filling)

**Figure 7:**
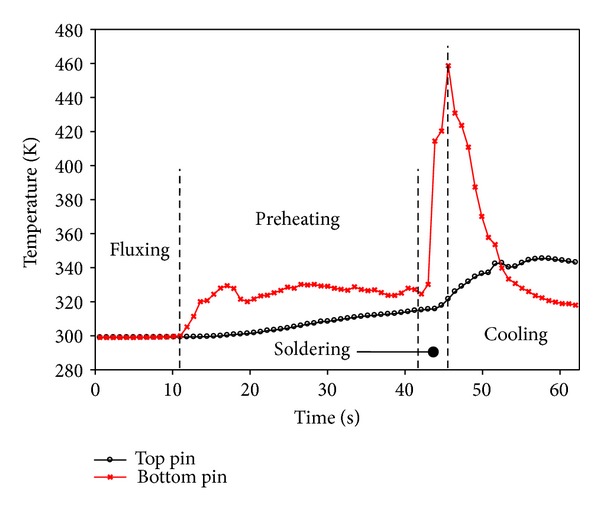
Temperature profiles at different zones.

**Figure 8:**
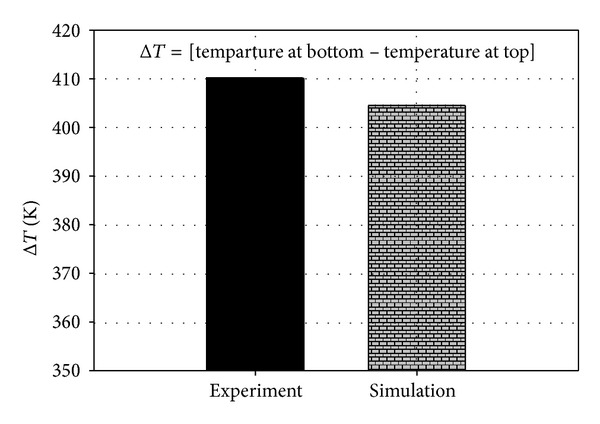
Comparison of Δ*T* between experiment and simulation.

**Figure 9:**
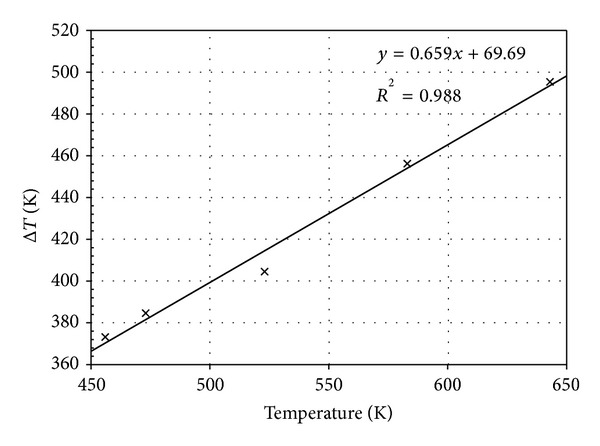
Δ*T* at different solder temperatures.

The experiment was performed to measure the temperature of the top and the bottom regions of the pin. During wave soldering, the bottom region of the pin was immersed in the molten solder as the PCB was passed through.  Figure 7 shows the temperature profile at different zones of the soldering machine. At the fluxing zone, both thermocouple temperatures were similar. The temperatures of the PCB and the pin increased when the preheating zone was reached. Subsequently, the PCB passed through the soldering zone as the capillary flow filled the PCB hole. At this instance, the pin reached a high temperature, whereas the temperature of the bottom pin region sharply decreased at the cooling zone. The temperature difference between the top and the bottom pin regions was calculated. The temperature difference was also estimated to obtain the predicted simulation result. The results are compared in Figure 8. The accurate predicted temperature could yield reliable results in the structural analysis. This is because the structural displacement and stress are corresponding to the temperature of molten solder. In the simulation, Δ*T* showed a linear correlation with the solder temperature, as illustrated in Figure 9. The simulation result was substantiated by the experimental measurement with a discrepancy as low as 1.37%. Thus, the excellent efficiency of the proposed thermal-FSI technique was demonstrated.

### 5.2. Overview of Thermal-Fluid Structure Interaction at *T* = 523 K

**Figure 10:**
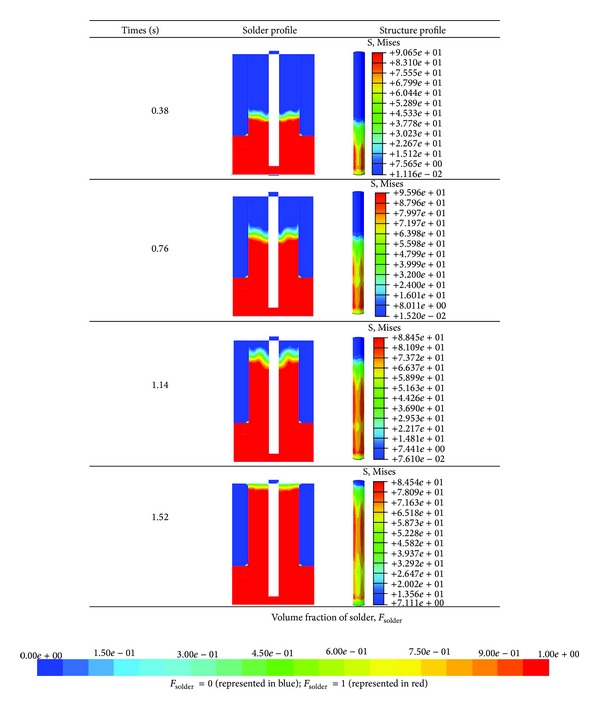
Overview of thermal-FSI.

In wave soldering, the molten solder fills the PCB hole through capillary action. Interactions between the molten solder (high temperature) and the pin cause displacement and stress. Therefore, an overview of the current predicted results was presented for a 523.15 K solder temperature. Generally, the pin is subjected to thermal stress (temperature-induced stress) when it is due to the restriction of thermal expansion at high temperature. As illustrated in Figure 4, the pin is fixed at the top surface, which is connected to the component. This boundary condition was defined based on the actual pin component, whereas the pin inserted into the PCB hole is restricted by the component (e.g., 2-pin resistor, capacitor, and multipin connector).  Figure 10 depicts that the solder profile and the von Mises stress imposed on the pin varied with filling time. The simulation results indicate the increase in the molten solder profile over time. This increase is attributed to the capillary action that induced the molten solder to fill the space as the PCB passed through the solder pot. The increase in the solder profile simultaneously increases the distribution of pin stress. This result indicates that the temperature of the molten solder contributes to the stress on the pin. This phenomenon is illustrated in Figure 10, which shows that pin stress corresponds to the profile of the molten solder.

### 5.3. Profiles at 50% and 100% Filling Levels

**Figure 11:**
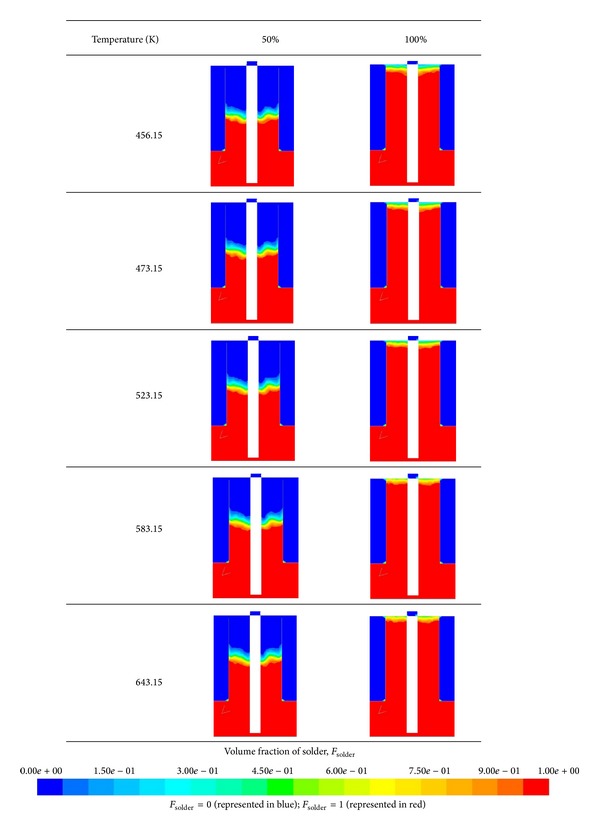
Filling profiles at 50% and 100% filling levels.

**Figure 12:**
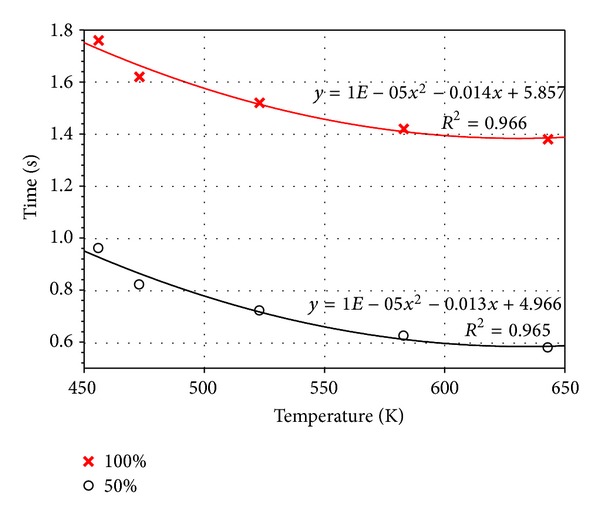
Filling times at 50% and 100% filling.

In the PCB assembly, the percentage of the PCB hole filling level is crucial to joint reliability. An uneven filling profile may cause incomplete filling and defects in the subsequent assembly process. Therefore, the filling profiles at varying solder temperatures were considered.  Figure 11 illustrates the solder profiles at 50% and 100% filling levels at different temperatures. At 50% filling level, the solder profiles were almost identical for all cases. Both left and right spaces exhibited a balanced flow front advancement. Despite the lack of significant variations in the solder profiles, the filling time differed for each case. The increase in solder temperature reduced the filling time, which may be attributed to the variations in molten solder viscosity at temperatures ranging from 456.15 K to 643.15 K. Solder viscosity decreased as the solder temperature increased, as indicated in Figure 12. Thus, the capillary flow easily filled the space, thereby reducing the filling time.

### 5.4. Temperature Distribution and Displacement on the PTH at 75% Filling

**Figure 13:**
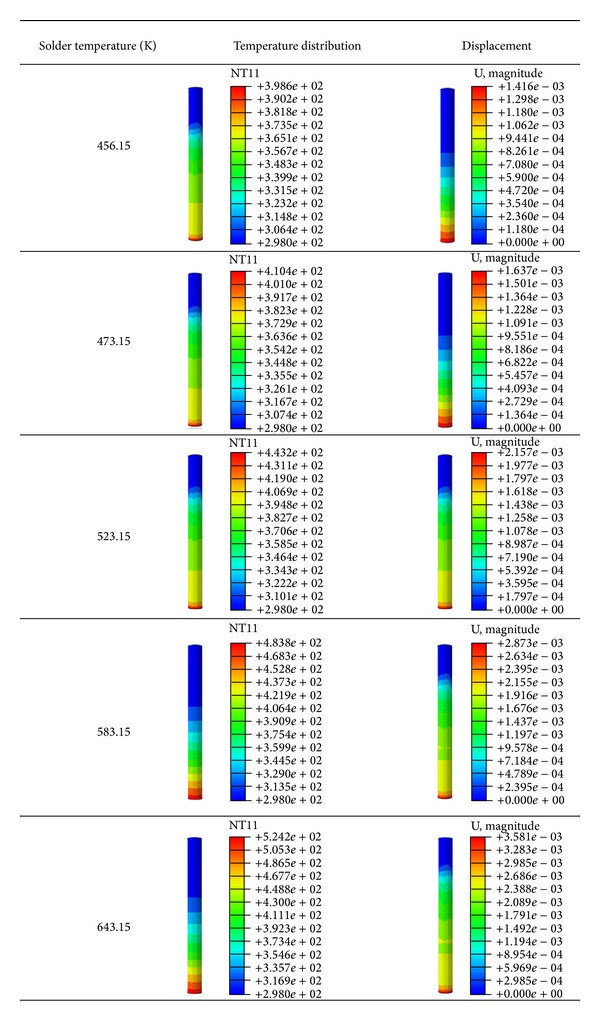
Temperature distribution and displacement at 75% filling.

**Figure 14:**
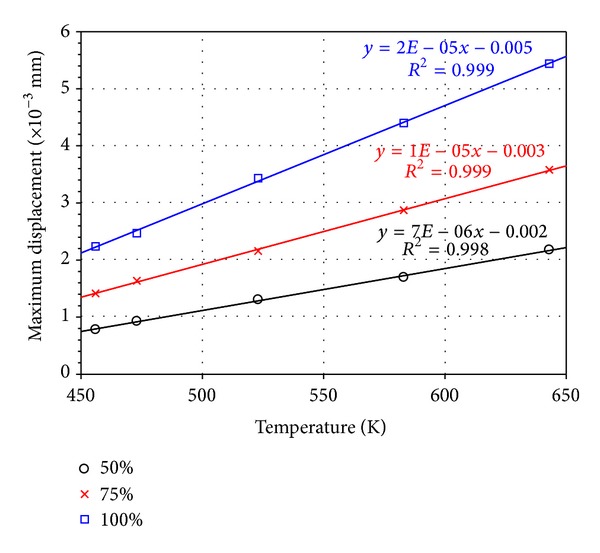
Maximum displacement of PTH at 50%, 75%, and 100% filling.

The temperature distribution and the displacement on the pin during capillary flow filling were predicted in the current thermal-FSI simulation. As mentioned in Section 5.2, temperature distribution corresponded to the solder profile and resulted in thermal stress. Moreover, pin displacement at a high molten solder temperature was evaluated at 75% filling. In this study, the displacement is referred to as the pin extension or deformation due to high temperature. To investigate the temperature effects, the low and extremely high temperatures were considered in the analysis, although it is not practicable in the industry. With this temperature range, the correlation of the temperature to the displacement and stress can be obtained from the simulation results.  Figure 13 shows the temperature and the displacement of the pin at varying solder temperatures. The highest temperature and the highest displacement were observed at the bottom end of the pin for all cases because this region endured the high molten solder temperature from the solder pot. Moreover, the pin conducted the heat from the molten solder during the filling process. Thus, the thermal effect resulted in pin displacement. A large pin displacement was concentrated at the bottom end; that is, the pin region exhibited increased temperatures during wave soldering. The variation of maximum pin displacement with solder temperatures at different filling levels is plotted in Figure 14. The pin displacement increases as the filling level increases. The pin conducts heat from the molten solder and then expands because of its thermal expansion properties. Hence, the pin is displaced from its initial position.

### 5.5. Stress Distribution at 50% and 100% Filling Levels

**Figure 15:**
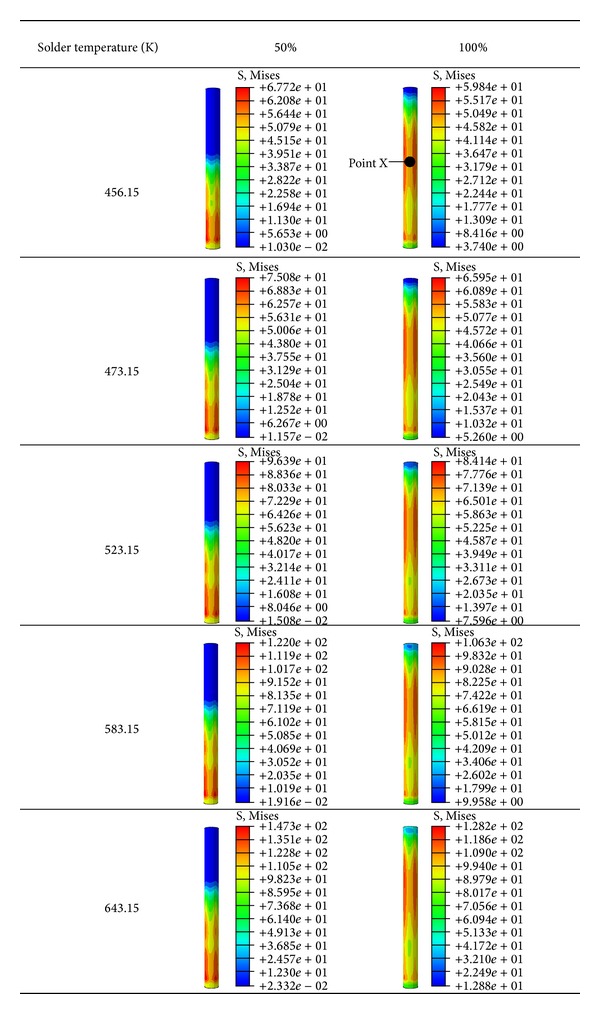
Stress distribution on pin.

**Figure 16:**
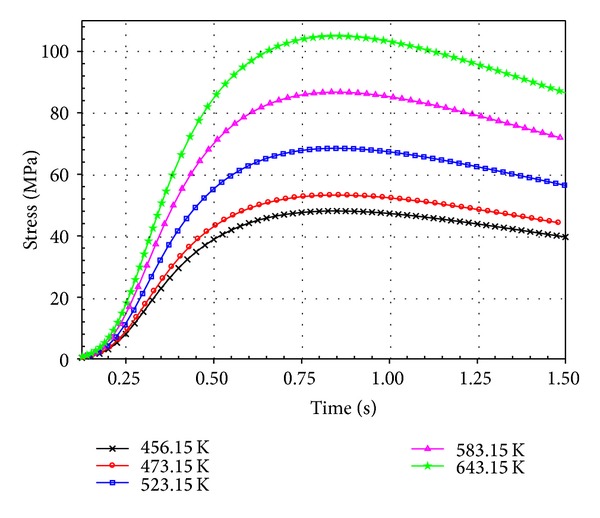
Stress distribution (MPa) on point X.

**Figure 17:**
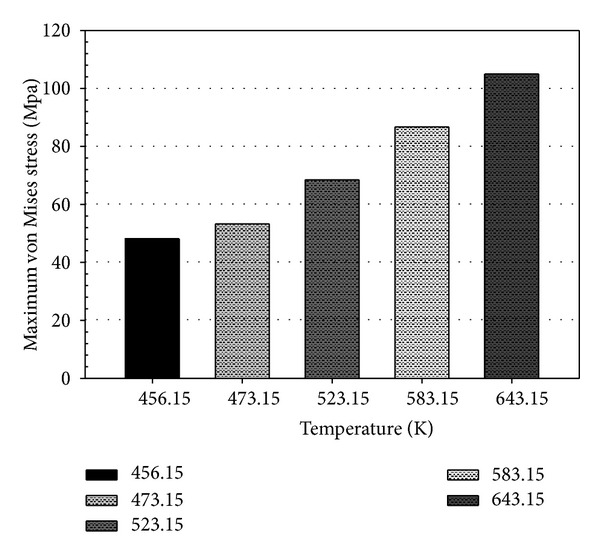
Maximum von Mises stress of PTH.

The stress profile of the pin at 50% and 100% filling levels was investigated.  Figure 15 illustrates the thermal stresses on the pin at temperatures ranging from 456.15 K to 643.15 K at 50% and 100% filling levels. At 50% filling, stress was concentrated on the bottom end of the pin. However, the highest stress was observed in the middle pin region located within the PCB hole. Moreover, the stress at point X (middle region of the pin within the PCB hole) was evaluated over the filling time, as plotted in Figure 16. High stress concentration may weaken solder joints and cause defects. Thus, pin stress considerably influences the joint reliability of the final product. The increase in solder temperature increases the maximum stress on the pin, as shown in Figure 17. Therefore, proper control of molten solder temperature is important to minimize the stress and displacement of the pin during wave soldering.

## 6. Conclusions

The effects of molten solder temperature on wave soldering were investigated using thermal-FSI modeling. Soldering temperatures ranging from 456.15 K to 643.15 K were considered in the study. The solder temperature exerted no significant effect on the solder profile. The capillary flow exhibited balanced flow front advancement during filling. However, the filling time exponentially decreased as the temperature increased. The temperature profile of the pin indicated that the pin reached the temperature of the molten solder during filling, which corresponded to the capillary flow. The bottom end of the pin endured the high temperature. During filling, the temperature of pin dropped in the vertical *z* direction. The heat conducted by the pin from the molten solder resulted in displacement and stress. At 643.15 K (100% filling), the pin was displaced by nearly 0.0055 mm and subjected to a maximum stress of 103 MPa. The high displacement of the pin also resulted from the conduction effect over a long filling period. This occurrence could weaken the joint between the pin and the PCB. Despite the lowest displacement (0.0022 mm) and stress (44 MPa) on the pin obtained at 456.15 K (100% filling), a longer filling time was required. Therefore, the soldering temperature should be optimized by considering other process parameters and physical designs to achieve the optimum joint reliability. Results revealed that filling time showed a quadratic behavior to the increment of temperature. The deformation of pin exhibited a linear correlation to the temperature. The predicted Δ*T* was verified by the experimental result. Thus, the excellent capability of the proposed thermal-FSI method in handling thermal fluid and structural analyses in wave soldering was demonstrated. The simulation results are useful for predicting trends and provide a better understanding of the wave soldering process for engineers in the electronics assembly industry.
